# Optical Fiber Sensors for the Detection of Hydrochloric Acid and Sea Water in Epoxy and Glass Fiber-Reinforced Polymer Composites

**DOI:** 10.3390/ma12030379

**Published:** 2019-01-25

**Authors:** Cristian Marro Bellot, Marco Sangermano, Massimo Olivero, Milena Salvo

**Affiliations:** 1 Department of Applied Science and Technology, Politecnico di Torino, c.so duca degli Abruzzi 24, 10129 Torino, Italy; cristian.marrobellot@polito.it (C.M.B.); milena.salvo@polito.it (M.S.); 2 Department of Electronics and Telecommunication, Politecnico di Torino, c.so duca degli Abruzzi 24, 10129 Torino, Italy; massimo.olivero@polito.it

**Keywords:** evanescent wave optical fiber sensors, diffusion, glass fiber-reinforced polymers, testing and aging

## Abstract

Optical fiber sensors (OFSs), which rely on evanescent wave sensing for the early detection of the diffusion of water and hydrochloric acid through glass fiber-reinforced polymers (GFRPs), have been developed and tested. Epoxy and GFRP specimens, in which these sensors were embedded, were subjected to tests in artificial sea water and hydrochloric acid. The sensors were able to detect the diffusion of chemicals through the epoxy and GFRP samples on the basis of a drop in the reflected signal from the tip of the optical sensor probe. Water and hydrochloric acid diffusion coefficients were calculated from gravimetric measurements and compared with the experimental response of the OFSs. Furthermore, mechanical tests were carried out to assess the influence of the sensors on the structural integrity of the GFRP specimens.

## 1. Introduction

The perfect combination of low cost, high corrosion resistance, high strength, easy manufacturing, and easy scalability has designated glass fiber-reinforced polymers (GFRPs) as promising materials for applications in hostile environments, such as underwater applications and the oil and gas industry [1]. GFRPs have been an important part of the fast-increasing expansion of petrochemical companies. However, there is still a lack of research on their failures and their aging is difficult to predict. For this reason, a great deal of effort has been devoted to embedding sensors into GFRPs for structural health monitoring purposes [2,3]. The aim of producing “sensitive” GFRP composites is to promote efficient maintenance programs with reduced costs, because any degradation (e.g., due to the diffusion of salty water or other chemicals in the polymer matrix) may be detected before the structural integrity is compromised. Optical fiber sensors (OFSs) can represent an excellent aid in such a framework, since they are minimally invasive and can operate remotely (for up to kilometers) without the need of any electrical supply.

In this paper, we focused on the design and experimental testing of low-cost OFSs for use in the monitoring of the diffusion of corrosive media through the thickness of epoxy resin and GFRP samples. OFSs can cope with the requirements of GFRPs for the oil and gas industry, because their small form factor and intrinsic fire safety make them very attractive, not just as temperature sensors (i.e., in wells during crude extraction) or as structural health monitoring probes [4], but also as chemical detection sensors [5].

The OFSs here described rely on the evanescent wave sensor concept developed by the authors and described in References [6,7]. In this work, the sensors were developed to selectively detect the diffusion of either salty water or hydrochloric acid (HCl), two of the several corrosion agents in the oil and gas industry [8]. Epoxy and GFRP samples were prepared by embedding OFSs, and diffusion was measured both by means of gravimetric measurements and by recording the response of the OFSs with a custom-developed multichannel spectroscopic system. The samples equipped with optical fiber sensors were also mechanically characterized and their properties were compared with pristine samples in order to evaluate how the embedded sensors could affect the mechanical properties of GFRP composites. 

## 2. Materials and Methods

### 2.1. Fabrication of Optical Fiber Sensors, Their Embedding into Epoxy and GFRP and the Interrogation Setup

The OFSs were fabricated from a commercial glass optical fiber (GIF 625 silica multimode fiber, Thorlabs). A 1.5-m-long optical fiber was cut from the spool for each sensor, and then one of the two tips was mechanically uncoated (i.e., the polymeric coating was removed by means of a stripping tool) and etched in hydrofluoric acid (HF) to remove the cladding, thus reducing the initial 125 µm fiber diameter to ~60 μm in order to expose the core—guiding the optical signal—to the surrounding environment. A reflective silver coating was then deposited onto the fiber tip by means of a quick chemical procedure based on Tollen’s reagent [9]. The thickness of the silver coating was ~5 µm. In this way, the optical signal traveling into the fiber core and containing the sensing information could propagate back to the interrogation unit, thus realizing a single-ended sensor [7]. The sensitive section of some of the sensors was specifically coated to make these sensors selective to HCl. Since HCl dissolves aluminum, these sensors (here referred to as Al-OFSs) were prepared by depositing a ~100-nm-thick aluminum coating on the bare core of the etched fiber by means of magnetron sputtering (Kenosistec, Plasma RF type).

**Figure 1:**
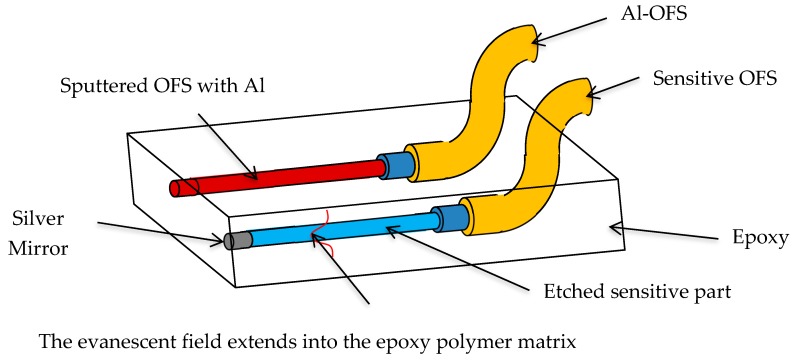
Scheme of an embedded optical fiber sensor (OFS) and sputtered OFS with aluminum (Al-OFS) in an epoxy polymer matrix (adapted from Reference [10]).

Sensitive epoxy specimens (with dimensions of 100 × 15 × 5 mm) were prepared by embedding the single-ended OFSs in the epoxy resin (Ampreg, Gurit, Switzerland) during the curing process (Figure 1). Details of the curing process are reported in Reference [7]. The sensors were embedded at different depths to detect chemical diffusion through the thickness of the polymer matrix and to evaluate the dynamics of the diffusion.

GFRP samples were prepared, following a similar workflow, by means of vacuum bag molding infusion, using the same kind of epoxy resin that was used for the epoxy samples, and then introducing an Advantex E-CR glass fiber fabric (Owens Corning, USA). The glass fiber plies were laid at orientations of 0° and 90°; three optical fiber sensors were deposited on the ply with an inter-distance of 0.5 cm. A peel ply and a diffusion mesh were put on top of the glass fiber plies. A plastic foil seal was used to cover all the plies and butyl tape was used to seal the infusion. The epoxy resin was mixed at a ratio of 100:33 and degassed, as described in Reference [7]. Finally, it was infused and left for 24 h at room temperature (r.t.) before proceeding with the thermal curing at 80 °C for 5 h.

**Figure 2:**
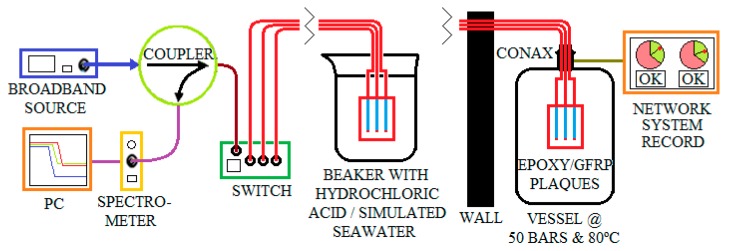
Interrogation setup. The inset shows the arrangement for high-temperature/high-pressure experiments (adapted from Reference [7]).

The sensors were monitored by means of the setup shown in Figure 2. Each sensor is optically supplied by a broadband source (Photonetics 3626BT), which emits a total maximum power of 25 mW over the 1500–1600 nm range. A fiber optics switch (JDS Uniphase 2 × 16 SB series) enables up to 16 sensors to be swapped; these sensors are connected to the switch by movable connectors (Thorlabs BFT-1). A fiber optic coupler separates the signal back-reflected from the sensor (which contains the sensing information) from the transmitted signal. The signal is processed and displayed on a portable spectrometer (Avantes AvaSpec-NIR256-1.7), which is able to measure spectra over the 1100–1700 nm range. The measured signal is compensated for by the background noise of the spectrometer and the spurious coupling between the source and the spectrometer. The system was designed for automatic switching among the sensors and logging of the spectra through a LabVIEW custom-developed program. The spectra may be recorded with a selectable sampling rate, and most of the experiments were carried out by recording spectra every 5 min, lasting from a number of hours to days. The inset in Figure 2 shows the arrangement of the experiments at high pressure (50 bars). In this case, the vessel is placed in a separate room for safety reasons. The fibers, which cross the wall between the two rooms, run through a Conax adaptor sealant. This adaptor has two holes in which up to four optical fibers per hole can be inserted. The adaptor was screwed directly onto the vessel, into which N_2_ air gas was pumped. Moreover, the vessel was connected to a network system in order to monitor the pressure and temperature during the experiments.

The diffusion coefficients were evaluated through gravimetric measurements (10 samples per test) for artificial sea water (according to ASTM D1141-98(2013) [11]) at 80 °C and at 80 °C/50 bars, as well as for HCl (37% Carlo Erba Reagents) at room temperature. The diffusion coefficient was calculated from the absorption curves, i.e., weight gain versus time, assuming, as an approximation, an ideal Fickian behavior using Equation (1) [12]:

(1) $$D=\;\pi {\left(\frac{h}{4M_{\infty }}\right)}^{2}{\left(\frac{M_{2}-M_{1}}{\sqrt{t_{2}}-\sqrt{t_{1}}}\right)}^{2}$$

where *h* is the sample thickness, $M_{\infty }$ is the weight gain at saturation, $M_{1}$ and $M_{2}$ are two experimental values on the linear section of the weight gain curve, and $t_{1}$ and $t_{2}$ are the corresponding time spots in s.

The expected diffusion length of the chemicals in the epoxy and GFRP was evaluated assuming Fick’s model and considering constant diffusion coefficients (Equation (2)):

(2) $$d=2\sqrt{Dt}$$

where *d* is the diffusion length, *t* is the time at which the chemical reaches length *d*, and *D* is the diffusion constant.

Furthermore, the real part of the refractive index was measured with a refractometer (Metricon 2010/M, New Jersey, USA) on pristine and water-saturated epoxy samples.

### 2.2. Mechanical Characterization of GFRP

A tensile test and a three-point bending tests were carried out on the GFRPs, with and without embedded sensors, to assess their mechanical properties and investigate whether and how embedded OFSs could affect the composite. Two sets of samples, that is, 10 without OFSs and 10 with three embedded OFSs, were prepared. The distance between the embedded optical sensors was ~1 cm.

The tensile test was carried out according to the ASTM D3039/D3039M-17 standard [13] using a mechanical testing machine (Zwick 750, ZwickRoell GmbH and Co., Ulm, Germany). Samples (250 mm × 25 mm × 2 mm) were arranged in the middle of holding grips, which had a gauge length of 50 mm. The load was applied by moving the cross-head at a speed of 2 mm/min.

Samples (80 mm × 15 mm × 2 mm) subjected to three-point bending were tested according to the ISO 14125 standard [14] using a mechanical testing machine (Zwick 100, ZwickRoell GmbH and Co., Ulm, Germany). The load was applied by moving the cross-head at a speed of 1.5 mm/min.

## 3. Results and Discussion

### 3.1. Mechanical Properties

In order to demonstrate that the embedding of the OFSs does not affect the mechanical properties of the composites, the GFRPs were mechanically tested with and without embedded OFSs. The results of the tensile and three-point bending tests are summarized in Table 1. The results confirm that the optical fibers have no relevant effects on the mechanical properties of the composites and are in agreement with early studies [15], though attention should be paid if the composites are subjected to a fatigue load. Furthermore, more specific mechanical characterizations should be carried out to assess the possible effect of embedded OFSs on the final component properties.

### 3.2. Characterization of Epoxy and GFRPs with Embedded OFSs in Artificial Sea Water at 80 °C

#### 3.2.1. Gravimetric Measurements and Monitoring of Diffusion by Means of the Optical Fiber Sensors

**Figure 3:**
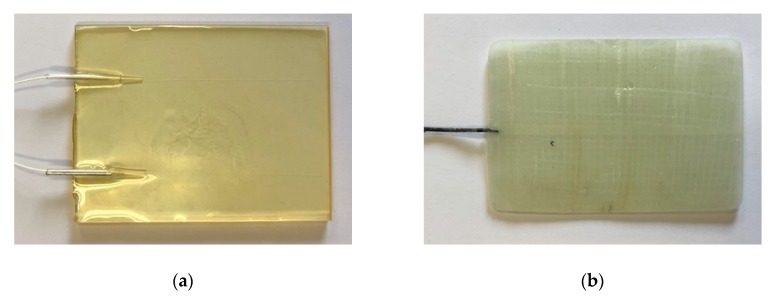
(**a**) Epoxy and (**b**) GFRP sample with embedded OFSs on the left-hand side.

OFSs were prepared and embedded in the epoxy matrix and in GFRP, as reported in Section 2.1. Figure 3a,b show an epoxy and a GFRP sample, respectively.

**Figure 4:**
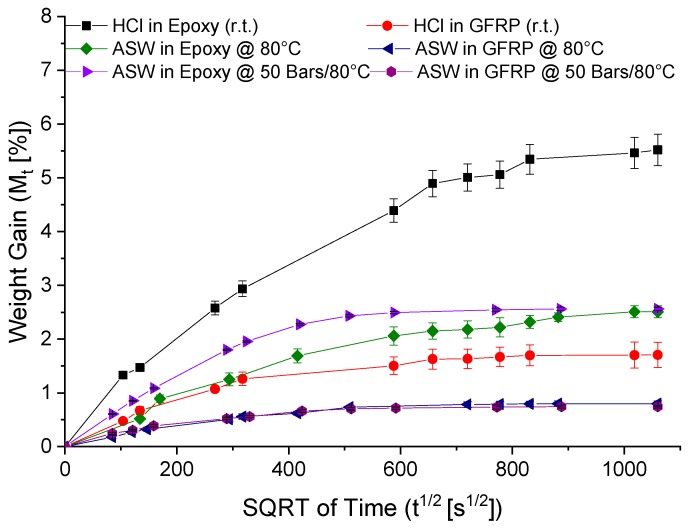
Weight increment of the epoxy and GFRP samples immersed in artificial sea water (ASW) at 80 °C, 80 °C under 50 bars of pressure and in HCl at room temperature.

The diffusion rates for the epoxy resin and GFRP were evaluated gravimetrically by immersing the samples into artificial sea water (ASW) under two different conditions: At 80 °C and at 80 °C under 50 bars of pressure. The diffusion values were also evaluated for the same samples, but this time immersed in HCl at room temperature. Figure 4 reports the weight gain versus time; the calculated diffusion coefficients are reported in Table 2. The diffusion coefficients were used to predict the diffusion time at a given depth using Equation (2). It is apparent from this table that the hydrostatic pressure affects the diffusion coefficient of water at 80 °C. Several studies have attempted to clarify the effect of hydrostatic pressure on water diffusion in composite materials [16,17,18], but the published results are contradictory. However, it is difficult to foresee, in general terms, the role of hydrostatic pressure, since the overall effect is due to a balance between the free-volume reduction (due to the increasing compression forces on the composites) and the increase of the absorption driving force (due to the increasing chemical potential in the liquid phase). In accordance with our study, Choqueuse at al. [17] showed that increasing the pressure results in faster water diffusion rates for epoxy-based materials.

The epoxy and GFRP samples containing the embedded sensors were immersed in artificial sea water heated to 80 °C for 96 h. The reflected signal from the sensors was recorded every 5 min over a spectral range of between 1500 and 1600 nm. The recorded spectra did not show any change in shape, whereas a drop in intensity was observed and ascribed to water diffusion. As discussed in Reference [7], the spectral attenuation is probably due to a change in the optical properties, particularly in the absorption coefficient of the epoxy surrounding the exposed core. The absorption coefficient represents the imaginary part of the refractive index and it affects the attenuation of the sensing region of the embedded optical fiber. In previous works (e.g., References [6,19]), it was demonstrated that the increment of absorption in the infrared region of the spectrum, observed after water diffusion, could be ascribed to OH stretching. Furthermore, the real part of the refractive index measured on pristine and water-saturated samples was about 1.551 without a remarkable variation caused by water exposure, hence concluding that the attenuation of the optical signal is caused by an increment of the optical absorption of the epoxy in the recorded spectral range. The maximum intensity of the signal provided by the source occurred at 1532 nm; hence, only this wavelength was analyzed in long-term experiments, considering it as a satisfactory representation of the entire spectrum.

**Figure 5:**
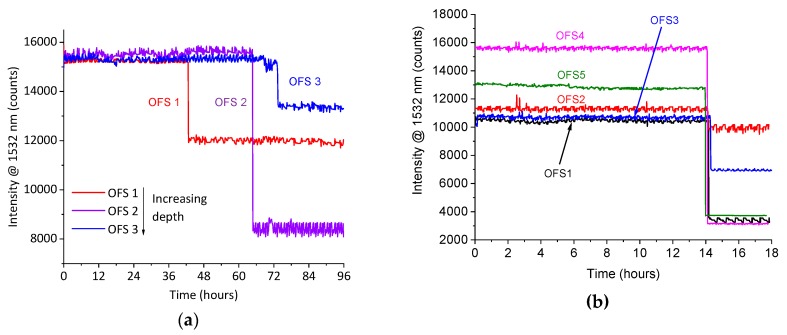
Signal intensity, at a wavelength of 1532 nm, from optical glass sensors embedded in: (**a**) Epoxy at different depths and (**b**) GFRP samples. The samples were immersed in artificial sea water at 80 °C.

Figure 5a shows the signal intensity at 1532 nm recorded from three different OFSs embedded in the epoxy at different distances from the surface. The signals from the optical sensors dropped at different times according to the depths of the sensors. The first sensor, located at a depth of 2.5 ± 0.1 mm, showed a drop in the signal after approximately 43 h. The second sensor, located at a depth of 2.8 ± 0.1 mm, showed a drop in the signal after approximately 64 h. Finally, the third sensor, located at a depth of 3.0 ± 0.1 mm, showed a drop in the signal after approximately 73 h. The calculated diffusion times, related to the depth of the three sensors, were 44, 63 and 72 ± 1 h, respectively. Hence, the OFSs were able to detect the water diffusion through the epoxy thickness with a very good time accuracy, in accordance with the calculated diffusion time.

Figure 5b shows the signal intensity drop at 1532 nm from five different OFSs embedded in GFRP, all of which were located at a distance of 0.9 ± 0.1 mm from the surface. As they were positioned at the same depth, the drop in the signals occurred at nearly the same time, i.e., after approximately 14 h. Assuming the approximation of a Fickian behavior, and using the diffusion coefficient reported in Table 2, the diffusion time for a thickness of 0.9 mm was 13 ± 1 h. This result shows that the OFSs also detected the water diffusion with a good accuracy when embedded in the composite.

#### 3.2.2. High-Pressure Experiments

In order to investigate the use of the OFSs under harsh conditions, bare OFSs were subjected to high pressures. The reflected signal was continuously monitored by exposing the OFSs to pressures ranging from 1 up to 75 bars, with a ramp pressure of 5 bars per minute. No degradation of the response of the sensors was observed, thus showing that the OFSs could withstand high-pressure conditions.

**Figure 6:**
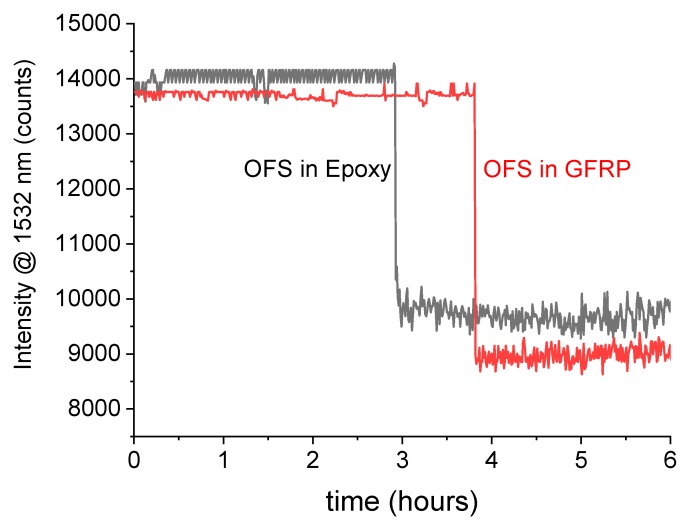
Signal intensity at a wavelength of 1532 nm from an OFS embedded in an epoxy and in a GFRP at depths of 0.9 ± 0.1 mm and 0.8 ± 0.1 mm, respectively. The samples were immersed in artificial sea water at 80 °C and 50 bars.

On the basis of this preliminary test, epoxy and GFRP samples containing OFSs were tested in artificial sea water at 80 °C under a pressure of 50 bars. The response of an OFS embedded in epoxy is reported in Figure 6 as an example. In this case, the depth of the OFS, as measured with a microscope, was 0.9 ± 0.1 mm. The reflected light intensity at 1532 nm showed a sharp decrease after around 3 h. This time was in accordance with the theoretical diffusion time calculated to reach a depth of 1.1 mm. Figure 6 also reports the signal recorded from an OFS embedded in GFRP and a drop was observed after about 4 h. For this diffusion time, the expected diffusion depth was about 0.7 mm. The response of the OFSs in these tests are in rough agreement with the expected diffusion time, yet they prove the capability of non-destructive, real-time detection.

### 3.3. Hydrocloric Acid Detection

OFSs, conceived to be selectively sensitive to HCl, were fabricated as described in Section 2.1, but the exposed core was then coated with a ~100-nm layer of aluminum. These sensors are here referred to as Al-OFSs.

**Figure 7:**
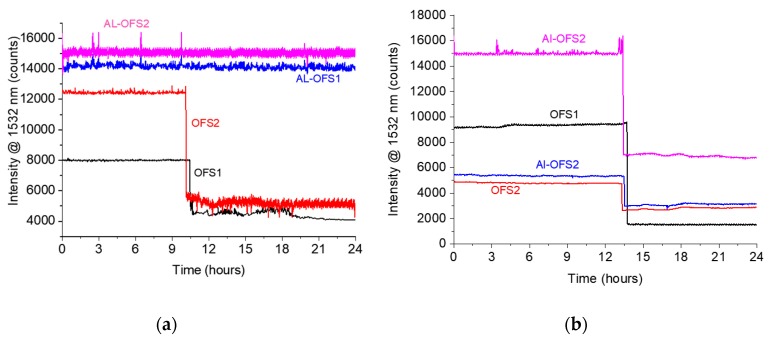
Signal intensity at a wavelength of 1532 nm from sensors embedded in the epoxy exposed to (**a**) artificial sea water and (**b**) HCl. The aluminum optical fiber sensors (Al-OFS) are sensitive to HCl, whereas they do not exhibit any response to artificial sea water.

The calculated diffusion coefficients of HCl at room temperature in the pristine epoxy were measured by means of a gravimetric test, as reported in Figure 4 and Table 2. Furthermore, epoxy samples containing both standard OFSs and Al-OFSs were immersed in artificial sea water and HCl, respectively, at room temperature, for up to 24 h. The results of the diffusion experiment are shown in Figure 7. Figure 7a reports the signal intensity recorded at 1532 nm, as a function of time, for the sensitive epoxy sample immersed in artificial sea water. On the other hand, Figure 7b depicts the outcome for the sensitive sample subjected to HCl. It can be observed that the standard OFSs detected both water and HCl diffusion, whereas the Al-OFSs were only responsive to HCL, since HCl can solubilize the thin Al-layer, thus enabling the core to be sensitive to changes in the refractive index and absorption of the epoxy. This is a proof-of-concept that the integration of OFSs and Al-OFSs can be exploited to selectively detect the diffusion of water and acid.

**Figure 8:**
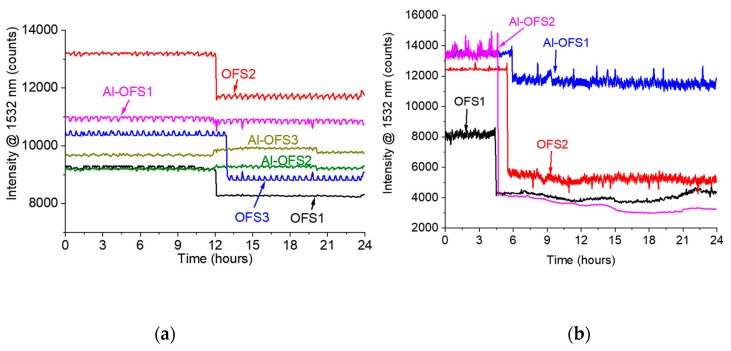
Signal intensity at a wavelength of 1532 nm from sensors embedded in GFRP exposed to (**a**) artificial sea water and (**b**) HCl. The Al-OFS are sensitive to HCl, whereas they do not exhibit any response to artificial sea water.

Analogous experiments were then performed with GFRP samples. Figure 8 reports the response of two sensitive samples exposed to artificial sea water and HCl at room temperature. While the signal drop was not the same for all the sensors, they exhibited the same behavior as those in epoxy. The standard OFSs detected the diffusion of both water and HCl, whereas the Al-OFS were only effective in sensing the diffusion of HCl. The time response was once more in agreement with that expected from Fick’s diffusion model.

The fabrication process reproducibility of these sensors still demands some improvements, as can be observed by comparing the initial and final intensity levels of the signals collected from the different experiments (Figure 5, Figure 6, Figure 7 and Figure 8). The initial reflectivity (i.e., the initial recorded signal) is strongly dependent on the quality of the mirror realized on the fiber’s tip, as well as on the connection to the interrogation unit. Furthermore, the variation observed on the final levels could be ascribed to the effective length of the sensing region, which is not perfectly controlled during fabrication.

## 4. Conclusions

Novel, single-ended, optical fiber sensors for the detection of chemicals have been fabricated, embedded, and tested in an epoxy resin and glass fiber-reinforced polymers. These sensors, which rely on evanescent field sensing, have the additional advantages of being single-ended and of being able to work as probes. A procedure to embed the optical fiber sensors has been developed and experimentally optimized. A setup for the continuous monitoring of the sensors has been designed to remotely track the embedded sensors. The recorded optical data of samples immersed in artificial sea water at 80 °C showed that it is possible to detect water diffusion by observing decreases in the spectral reflection from the sensors. The results were then corroborated by means of gravimetric measurements and a Fickian diffusion model. Tests conducted for up to 100 hours showed that the response of the sensors embedded at increasing depths in the samples was in full agreement with the expected diffusion time. Experiments at high pressure, carried out in a special vessel, also exhibited acceptable agreement between the experimental data and the calculated diffusion time. Furthermore, tests at high pressure yielded some knowledge about the installation of optical fiber sensors in harsh environments and at hard-to-reach locations.

By tailoring the sensors, it was possible to selectively detect the diffusion of hydrochloric acid. To implement this feature, the standard fabrication process was upgraded by coating the etched fiber with a 100-nm aluminum layer, which reacted to the hydrochloric acid.

Embedding optical glass fibers in the GFRP composites did not influence their mechanical properties, as shown by means of a tensile test and a three-point bending test.

In short, this research has provided a proof-of-concept of a sensitive GFRP composite that could be used, for example, in a pipeline to monitor the diffusion of chemicals in real time and provide information in order to reduce maintenance services.

## 5. Patents

M. Salvo, M. Sangermano, C. Marro Bellot, M. Olivero, Optical Sensor, Process for Making Such Sensors and Evaluation System Comprising at Least One of Such Sensors, 2018WO–IT00059, 2017.

## Figures and Tables

**Table 1:** Mechanical test results of the tensile test and three-point bending test of GFRPs with and without embedded OFSs.

	Tensile Test		Three-Point Bending Test	
	σ (MPa)	E (GPa)	σ (MPa)	E (GPa)
GFRP	367 ± 15	22 ± 5	364 ± 24	17 ± 1
GFRP + OFSs	356 ± 23	20 ± 3	368 ± 26	17 ± 1

**Table 2:** Diffusion coefficients calculated from Figure 4. ASW = artificial sea water.

	Epoxy	GFRP
**ASW 80 °C**	$8.8\pm 0.4\times 10^{-12}\;m^{2}/s$ [7]	$4.9\pm 0.1\times 10^{-12}\;m^{2}/s$
**ASW 50 bars/80 °C**	$2.9\pm 0.1\times 10^{-11}\;m^{2}/s$	$8.9\pm 0.1\times 10^{-12}\;m^{2}/s$
**HCl r.t.**	$1.4\pm 0.4\times 10^{-11}\;m^{2}/s$	$4.5\pm 0.2\times 10^{-12}\;m^{2}/s$
